# An Actual Natural Setting Improves Mood Better Than Its Virtual Counterpart: A Meta-Analysis of Experimental Data

**DOI:** 10.3389/fpsyg.2020.02200

**Published:** 2020-09-30

**Authors:** Matthew H. E. M. Browning, Nathan Shipley, Olivia McAnirlin, Douglas Becker, Chia-Pin Yu, Terry Hartig, Angel M. Dzhambov

**Affiliations:** ^1^Virtual Reality and Nature Lab, Department of Parks, Recreation and Tourism Management, Clemson University, Clemson, SC, United States; ^2^Department of Natural Resources and Environmental Science, University of Illinois at Urbana-Champaign, Champaign, IL, United States; ^3^Department of Environmental Health, Harvard T.H. Chan School of Public Health, Boston, MA, United States; ^4^School of Forestry and Resource Conservation, National Taiwan University, Taipei, Taiwan; ^5^Institute for Housing and Urban Research, Uppsala University, Uppsala, Sweden; ^6^Department of Psychology, Uppsala University, Uppsala, Sweden; ^7^Department of Hygiene and Ecomedicine, Faculty of Public Health, Medical University of Plovdiv, Plovdiv, Bulgaria

**Keywords:** green space, virtual reality, emotion, mental health, environmental simulations, restorative environments, systematic review

## Abstract

Accumulating evidence indicates that simulated natural settings can engage mechanisms that promote health. Simulations offer alternatives to actual natural settings for populations unable to travel outdoors safely; however, few studies have contrasted the effects of simulations of natural settings to their actual outdoor counterparts. We compared the impacts of simulated and actual natural settings on positive and negative affect (mood) levels using a pooled sample of participants enrolled in extant experimental studies. Relevant articles were identified from a review of research published/in press by March 2020 and updated during the peer review of the current study. Of 16 articles identified, 6 met the inclusion criteria and administered a single cross-cutting, standardized instrument [the Positive and Negative Affect Schedule (PANAS)] before and after exposure. Random effects meta-analysis of pooled effects showed that positive affect increased in the actual settings but not in their simulated counterparts (Hedge's *g* = 0.87; 95% CI, 0.54, 1.20). We observed little difference in effects on negative affect change scores (*g* = −0.28; 95% CI, −0.62, 0.06), with studies generally showing reductions in negative affect in both settings. Further research with additional populations, settings, antecedent conditions, and durations would provide a more robust understanding of differences in effects between these two ways to enhance mood by viewing nature.

## Introduction

Health benefits of visits to natural settings are unavailable for many people. Urbanities often do not have ready access to public or private green space where they can recreate outdoors (Beyer et al., 2018; Haydock and Moran, 2019). Hospital patients, nursing home residents, physically disabled adults, and prison inmates spend even greater shares of their time indoors. Special circumstances, like the shelter-in-place orders issued during the 2020 COVID-19 pandemic, may restrict access to outdoor settings even for people who could otherwise enjoy them. Without access to natural settings, people may forgo myriad health benefits—from reduced mortality and diabetes rates to improved mental and cardiovascular function (Hartig et al., 2014, 2020; Twohig-Bennett and Jones, 2018).

Ample evidence suggests that nature simulations can, under some conditions, support processes that promote health. More than 100 experiments report that pictures, videos, or immersive virtual environments with natural elements boosted mood, enhanced executive cognitive functions, promoted physiological stress recovery or reduced pain with little to no side effects (Browning et al., 2020a). Yet, how and to what extent simulations replicate the benefits of actual natural settings remain essentially unknown. More knowledge in this regard would help research and practice communities to better understand the circumstances in which simulation-based interventions can and cannot offer benefits like those described in the broader nature-and-health field.

**Figure 1:**
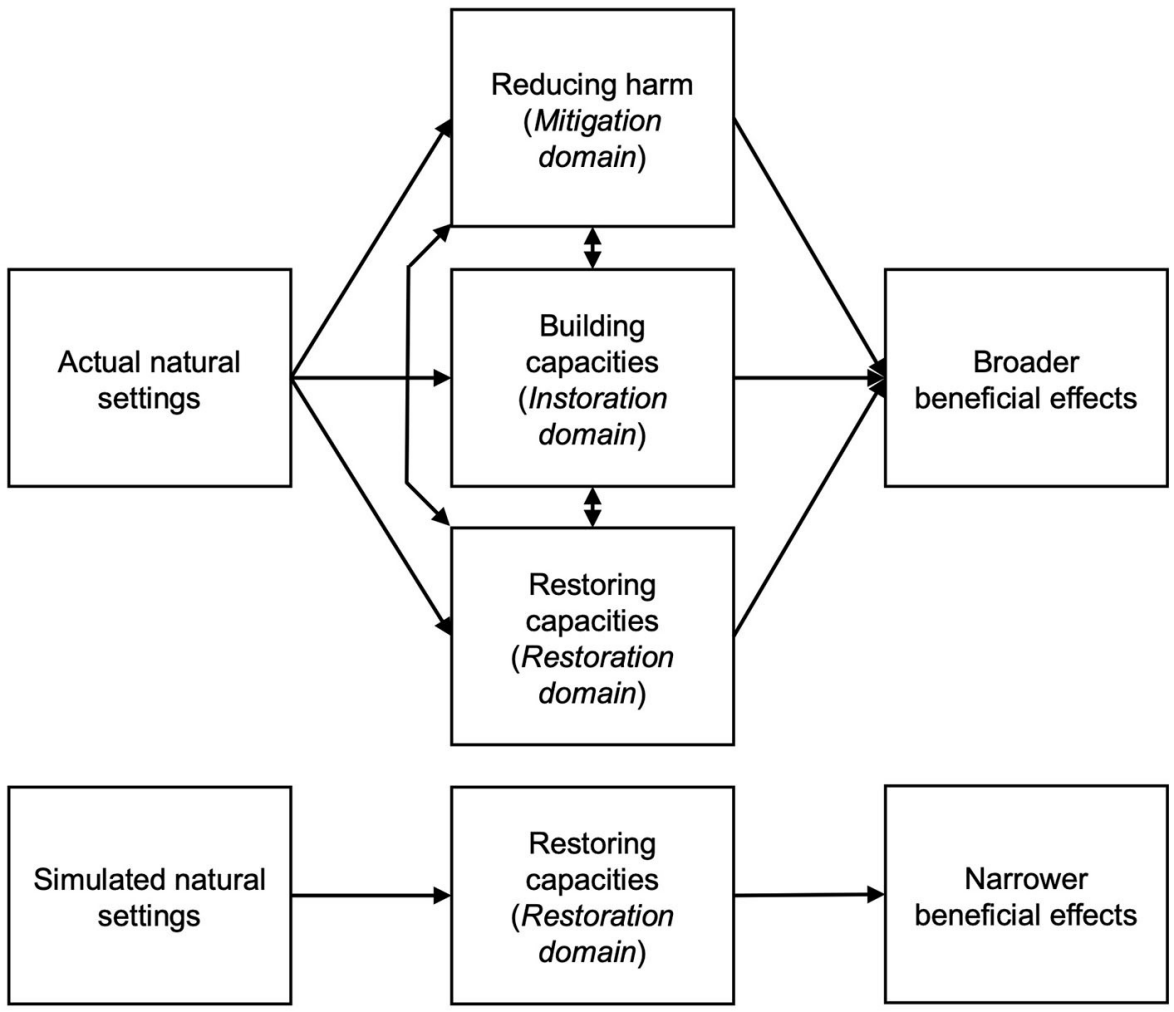
Actual natural settings may activate more pathway domains that promote beneficial health effects than do simulated natural settings. Model adapted from Markevych et al. (2017).

How might simulated natural settings yield benefits like those found with exposure to actual nature? Examination of the conceptual framework developed by a panel of experts on the health benefits of nature exposure is helpful for comparing the expected benefits from simulated and actual settings (Markevych et al., 2017). This framework explains three sets (domains) of pathways that explain the health benefits of natural settings, including reducing harm (the “mitigation” domain), restoring capacities (the “restoration” domain), and building capacities (the “instoration” domain). Both actual and simulated settings can support the renewal of depleted adaptive resources, as through stress recovery and directed attention restoration (pathways within the restoration domain). Other mechanisms may be less likely to be activated in simulations, including reducing air and noise pollution (mitigation pathways) and promoting physical activity and social contacts (instoration pathways) (see Figure 1). Simulations could activate mitigation and instoration pathways if they masked noise in loud environments (e.g., hemodialysis centers; Burrows et al., 2020), accompanied vigorous walking on treadmills or cycling on stationary trainers (Howard, 2017; Birenboim et al., 2019), or supported interactions between multiple users (White et al., 2018; Riva et al., 2020). However, the vast majority of simulations today offer passive single-person experiences with only audio input, only visual input, or a combination of the two (LaValle, 2017). They, therefore, presumably work primarily through restoration pathways, with restoration broadly conceived to include recovery from boredom and understimulation as well as from efforts to meet excessive demands (Ulrich, 1983; Frankenhaeuser and Johansson, 1986).

To our knowledge, only two reviews have examined the effects of simulated vs. actual exposure to nature, but their conclusions on this topic are limited (McMahan and Estes, 2015; Lahart et al., 2019). Few of the included experiments directly compared the effects of viewing the exact same setting in both a simulation and outdoors. Presenting the same setting in both experimental conditions (actual and simulated) strengthens the internal validity of results, in that differences in outcomes between conditions cannot be attributed to differences between the settings presented (Rossetti and Hurtubia, 2020).

Here, we employed a meta-analytical approach to compare the effects of actual and simulated natural settings on human health/well-being. Because this approach was applied to an emerging topic (Browning et al., 2020a), we conducted a review that included the greatest number of studies possible despite the likelihood that the number of eligible studies would be small. Accordingly, we aimed to provide a benchmark for the current state of evidence upon which future research can build, together with an initial framing of the research problem and articulation of relevant methodological issues.

## Methods

### Study Protocol

This meta-analysis originated from a systematic review conducted by some of the coauthors here, which is described elsewhere (Browning et al., 2020a). The review and this meta-analysis followed the Preferred Reporting Items for Systematic Reviews and Meta-analysis (PRISMA) guidelines (Moher et al., 2009).

### Conducting Study Protocol and Search Strategy

We identified the bulk of relevant papers by consulting the results of the former large systematic review by Browning et al. (2020a), in which the authors conducted an extensive keyword search in Scopus, PubMed, and Web of Science for articles that were published or in press by January, 2019 and that referenced natural settings and simulations in their titles, abstracts, or keywords (see Table S1 for list of keywords). Articles were included in that review if they met the following criteria: (A) participants had been exposed to at least one simulated natural setting, such as a photograph, slideshow, video, or immersive virtual environment (i.e., 360° video or computer-generated three-dimensional environment); (B) researchers measured at least one human health or cognitive performance outcome; and (C) researchers compared the results of different treatments using inferential statistics.

We then followed the methods from another meta-analysis on the effects of environmental exposure on human health to select which health/well-being outcome measure(s) to analyze (Radke et al., 2020). For selection, an outcome should show sensitivity to short-term exposure to natural settings that over time could cumulatively affect health in lasting ways. It should also indicate changes that could follow from either type of exposure (actual or simulated) and which would reflect the operation of any of the multiple pathways that could become engaged (instoration, restoration, and mitigation). Positive and negative affect (mood) met these criteria and were chosen to analyze. Stress reduction/buffering also met these criteria but were measured with disparate measures in the former systematic review, including self-report measures or indices and physiological measures, making meta-analyses not possible. Our selection of outcome (mood) also built on findings of the only review that examined the topic of exposure to simulated vs. actual nature and was published before the current study began (McMahan and Estes, 2015)^1^. All aspects of mood were considered—including affective arousal and valence or combinations of these—as long as attributes were measured with standardized self-report instruments with demonstrated construct validity, criterion validity, reliability, and sensitivity to change (Coste et al., 1997).

Next, we narrowed the sample of articles identified in the review by Browning et al. (2020a) to those that might be used to address our specific objectives. Three inclusion criteria were added: (A) researchers reported changes in mood before and after exposure to at least one simulated natural landscape using a standardized measure; (B) researchers employed a simulation of a natural setting that was the same—or very similar—to the actual setting used in the same study; and (C) exposures to the simulated and actual settings had similar durations.

To ensure that our results were comprehensive and up to date, we reviewed several other sources of data and published articles. First, we sought unpublished datasets to identify and overcome publication bias and increase the precision of our reported meta-effects (Dickersin et al., 1994; Card, 2015). Unpublished data were solicited with postings on five prominent scientific and professional listservs and emails to colleagues of the coauthors of the current study. Second, we included a supplemental keyword search for dissertations and theses using ProQuest. These types of reports can contain valuable data on emerging areas of research (Card, 2015). Third, we examined the citations of two narrative reviews. One considered the health benefits of simulated natural settings in virtual reality (White et al., 2018). The other reviewed experiments that tested the transferability of findings from laboratory simulation studies to actual *in situ* field studies (Rossetti and Hurtubia, 2020).

### Extracting Data

Article identification and data extraction were independently performed by two of the study authors. Disagreements were resolved through discussion among three members of the research team attending to data extraction. The interrater reliability was 100% agreement (*k* = 1.0) (Belur et al., 2018). Codes for article inclusion/exclusion and data from included articles were entered into a standard data extraction spreadsheet in Microsoft Excel for Mac (Redmond, WA, USA). Variables extracted are covered in the next section.

### Analyzing Data

We compared mood changes using standardized mean difference scores (Higgins and Green, 2011b; Card, 2015). These scores were calculated using the mean difference divided by the standard deviation (Higgins and Green, 2011b). Mean difference scores were calculated as the mean change (postexposure mean minus the preexposure mean) for the actual setting minus the mean change for the simulated setting. The standard deviations were calculated using the formula provided in the Cochrane Handbook for Systematic Reviews of Interventions (Higgins and Green, 2011b):

$$\begin{array}{l} SD_{Change}\\ =\sqrt{SD_{Baseline}^{2}+SD_{Final}^{2}-\left(2\times Corr\;\times SD_{Baseline}\;\times SD_{Final}\right)} \end{array}$$

Here, *SD*_*Change*_ is the standard deviation of the change in one of the experimental conditions (actual or simulated nature). *SD*_*Baseline*_ is the standard deviation of the prescore, and *SD*_*Final*_ is the standard deviation of the postscore. *Corr* is the correlation between the pre- and postscores.

We pooled the data and estimated an overall effect size using a random-effects model fitted with maximum-likelihood estimation to capture both the sampling error and the between-study variability. As a sensitivity analysis, we also employed the inverse variance heterogeneity model (IVhet), which is believed to yield more conservative effect estimates (Doi et al., 2017). The mean effect was expressed as a standardized Hedge's *g*, which is a less biased measure than Cohen's *d* for the small number of samples that we expected in this emerging research topic (Rosenthal, 2009; Card, 2015). Values below 0.2 represent a small effect size, below 0.5 represent a medium effect size, and values above 0.8 represent a large effect size (Hedges and Olkin, 2014). As a sensitivity analysis, we used the leave-one-out method to check the robustness of the pooled effect estimate after excluding the estimate from any given study (Dzhambov and Lercher, 2019).

Heterogeneity between study effect sizes was tested using Cochran's *Q* statistic and evaluated using the *I*^2^ statistic (Higgins and Green, 2011b). A significant *Q* statistic indicates that there is substantial heterogeneity between studies, and the *I*^2^ statistic helps interpret the proportion of overall variability that can be attributed to between-study heterogeneity. Values for *I*^2^ below 30 indicate that little total variability is attributable to between-study heterogeneity; values between 30 and 60 represent moderate levels of heterogeneity; and from 60 to 100, substantial levels (Higgins and Green, 2011a).

### Detecting Publication Bias

We employed the Doi plot for detection of publication bias (Furuya-Kanamori et al., 2018). Doi plots are variants of the normal quintile vs. effect plot—the former plots a rank-based measure of precision (Z score) instead of the standard error against effect size. Plot asymmetry was quantified with the Luis Furuya-Kanamori (LFK) index (Furuya-Kanamori et al., 2018, 2020). A symmetrical, mountain-like Doi plot and an LFK index <|1| indicate no asymmetry. An LFK index between |1| and |2| indicates minor asymmetry, and an LFK index >|2| indicates major asymmetry (Furuya-Kanamori et al., 2018).

### Evaluating Quality of Evidence

Our approach to evaluating methodological biases addressed the relevant domains in the Cochrane Collaboration's tool for assessing risk of bias in intervention studies (Higgins et al., 2011). These included random sequence generation, allocation concealment, blinding of participants and personnel, blinding of outcome assessment, incomplete outcome data, reporting bias, and other biases (see Table S2 for details). Each article received one of three scores for each domain: (1) low risk, which describes bias(es) that would be unlikely to alter the results seriously; (2) unclear risk, which describes bias(es) that raise some doubt about the results; or (3) high risk, which describes bias(es) that may alter the results seriously.

After bias evaluation, the quality of evidence across studies was synthesized to determine the strength of evidence for mood differences between actual and simulated natural settings. We employed a method that was adapted to the framework developed by Radke et al. (2020), which in turn was informed by the Grading of Recommendations, Assessment, Development, and Evaluations (GRADE) approach (Balshem et al., 2011). Radke et al. (2020) examined six attributes of associations between environmental exposure data and health outcomes that could be used to support causation: consistency, exposure–response relationships, strength of association, temporal relationship, biological plausibility, and coherence. Here, we considered only experimental studies with pretest–posttest designs and, therefore, selected only those additional attributes relevant to the current meta-analysis: consistency (similarity of results across studies) and strength of association (effect magnitude and precision of reported results).

After considering these attributes, the strength of evidence for the difference between each mood outcome under consideration was assigned a score of *Robust, Moderate, Slight, Indeterminate*, or *Compelling evidence for no effect*. The highest two categories describe evidence that strongly supports a difference in mood change between exposures. These two are differentiated by the quantity and quality of information available to rule out alternative explanations for the results. The middle two categories describe evidence for which uncertainties prevent drawing a conclusion in either direction. These categories are limited by low numbers of studies or substantial heterogeneity across studies. The final category describe a situation where several high confidence studies show null results.

### Software

Data analyses were conducted in R version 3.6.1 (Vienna, Austria). Effect size calculations, meta-analysis, and publication bias tests were conducted using the “metaphor” package version 2.1-0 (Viechtbauer, 2010). The IVhet meta-analysis and publication bias tests were conducted in MetaXL v. 5.3 (EpiGear International Pty Ltd, Sunrise Beach, Queensland, Australia).

## Results

### Study Selection

Twelve articles identified in the systematic review by Browning et al. (2020a) were relevant. Three more were identified through listserv postings, contact with colleagues, dissertation searches, and narrative reviews. One more was identified while the current study was in peer review. Of these 16 relevant studies, one was excluded because they varied the duration of treatments between actual and simulated conditions (Ryan et al., 2010). Seven were excluded because they did not use standardized measures of mood and/or were not designed to assess change in affect across a defined exposure (Hartig et al., 1997; Kahn et al., 2008; Huang, 2009; Mayer et al., 2009; Kjellgren and Buhrkall, 2010; Lassonde et al., 2012; Yin et al., 2018). Two more were excluded because they would have introduced substantial heterogeneity in models; one study used a unique mood measurement that contrasted with the bulk of the other included articles (Gatersleben and Andrews, 2013), and the other (Plante et al., 2006) measured an entirely different dimension of mood: activation rather than valence (Kensinger and Corkin, 2004).

**Figure 2:**
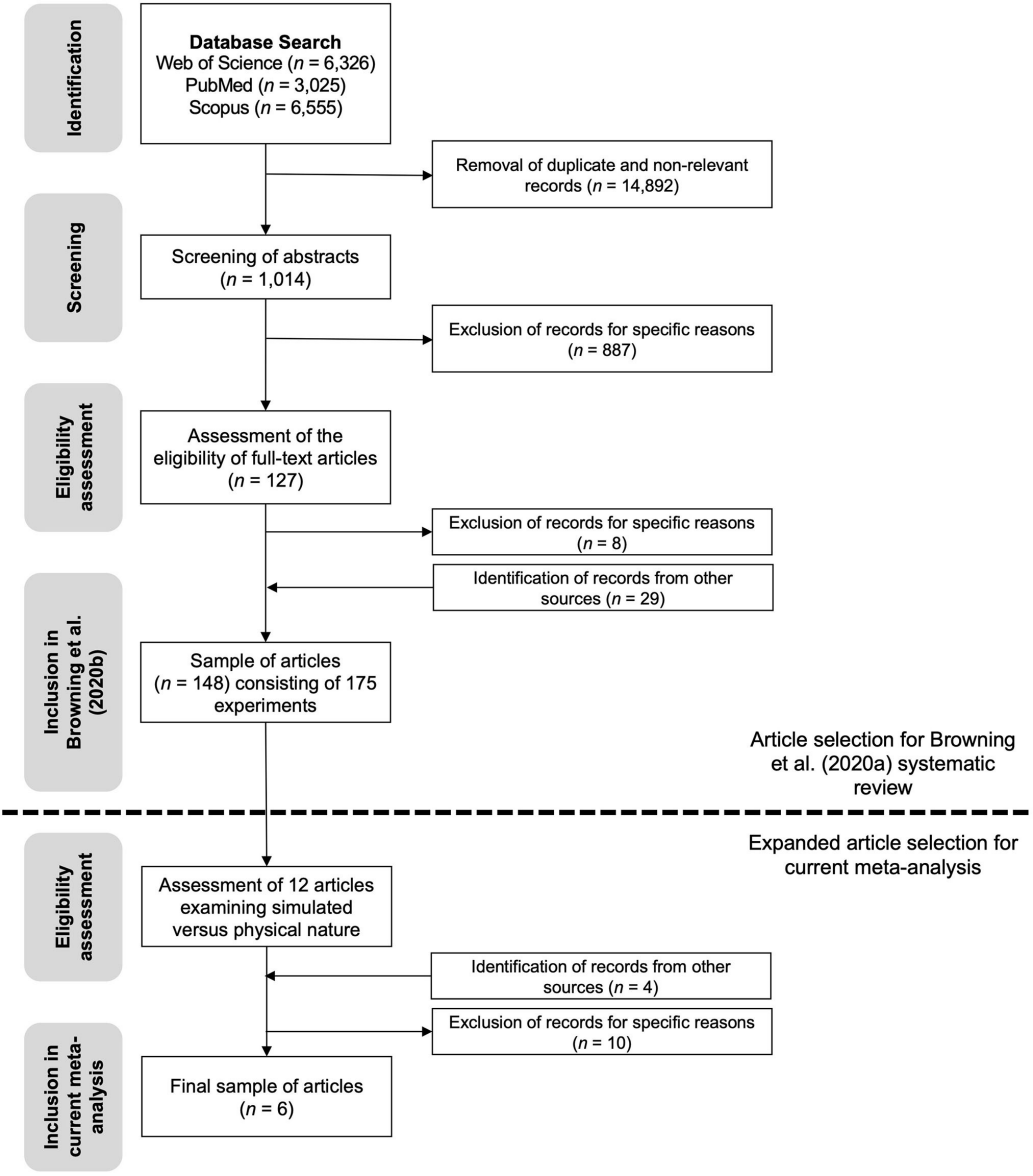
A flowchart of the process by which experiments were selected for inclusion in the systematic review by Browning et al. (2020a) as well as the current meta-analysis.

Our final sample consisted of six studies (Brooks et al., 2017; Calogiuri et al., 2018; Olafsdottir et al., 2018; Chirico and Gaggioli, 2019; Browning et al., 2020b; Nukarinen et al., 2020). All included studies used the same cross-cutting measure of mood—the Positive and Negative Affect Schedule (PANAS)—to measure changes in negative and positive affect levels. See Figure 2 for an overview of the process by which articles were identified and considered for inclusion.

### Study Characteristics

Table 1 shows the sample, study design, and simulation characteristics of the articles included in the meta-analysis. Samples consisted primarily of young adults with a pooled age of 24, weighted by sample size (standard deviation, 2.3). All studies were conducted in Global North countries and used relatively small sample sizes (24–82). Computer monitors were used in two studies, and head-mounted displays (HMDs) were used in four studies. HMDs can be used to project 360-videos of actual natural settings captured with fish-eye lenses cameras or computer-generated virtual environments (for a review of both techniques, see Browning et al., 2020c; Joseph et al., 2020). No study attempted to induce acute stress or attentional fatigue before the environmental exposure so that effects could more readily be understood as restorative. One of the HMD studies reported that 19 of 26 participants experienced cybersickness (Calogiuri et al., 2018). Cybersickness involves symptoms similar to those of motion sickness that can be caused either by vestibular stimulation (physical movement) or visual stimulation (observed movement) in HMDs (LaViola, 2000). Symptoms may include eye strain, headache, pallor, sweating, dryness of the mouth, fullness of the stomach, disorientation, vertigo, ataxia (lack of coordination), nausea, vomiting, dizziness, salivation, and burping (LaViola, 2000; Davis et al., 2014). No adverse effects were reported in other simulations.

**Table 1:** Characteristics of studies that met the inclusion criteria of the review.

**References**	***N***	**Age(M)**	**Female(%)**	**Country**	**Simulation experience**	**Simulationduration (min)**	**Natural setting**
Brooks et al. (2017), study 3	47	22	81	Canada	Sitting and watching pictures on computer screen	10	Relatively open landscapes during winter conditions covered with snow and blue skies with evergreen trees in the foreground and low mountains in the background (see p. 97 and Figure 2 in that article)
Calogiuri et al. (2018)^a^	26	26	46	Norway	Walking on treadmill and watching moving 360° video in an HMD	10	Paved trail along lake with brown grass and trees without leaves and partly cloudy blue skies (see pp. 4–5 and Figure 2 in that article)
Olafsdottir et al. (2018)^a^	67	24	69	Iceland	Walking on treadmill and watching moving video shown on television screen	40	Trail through forest dominated by evergreen trees with intermittent views of open natural landscapes including green spaces, moss-covered lava fields, and mountains (see pp. 7–8 and Figure 2 in that article)
Chirico and Gaggioli (2019)	50	24	50	Italy	Sitting and watching stationary 360° video in an HMD	5	Panoramic overlook of scenic lake with mountains (see p. 2 in that article)
Nukarinen et al. (2020)^a^	24	26	54	Finland	Sitting and watching stationary 360° video in an HMD	10	Forest on edge of lake (see p. 3 in that article)
Browning et al. (2020b)	82	20	48	United States	Sitting and watching stationary 360° video in an HMD	6	Moderately dense forest with deciduous trees with small bluff overlooking stream (see p. 13 in that article)

* *Three experimental conditions were tested in these studies. We included the effect estimates from the simulated condition that most closely resembled the actual nature condition. HMD, head-mounted display*.

### Synthesized Findings

**Figure 3:**
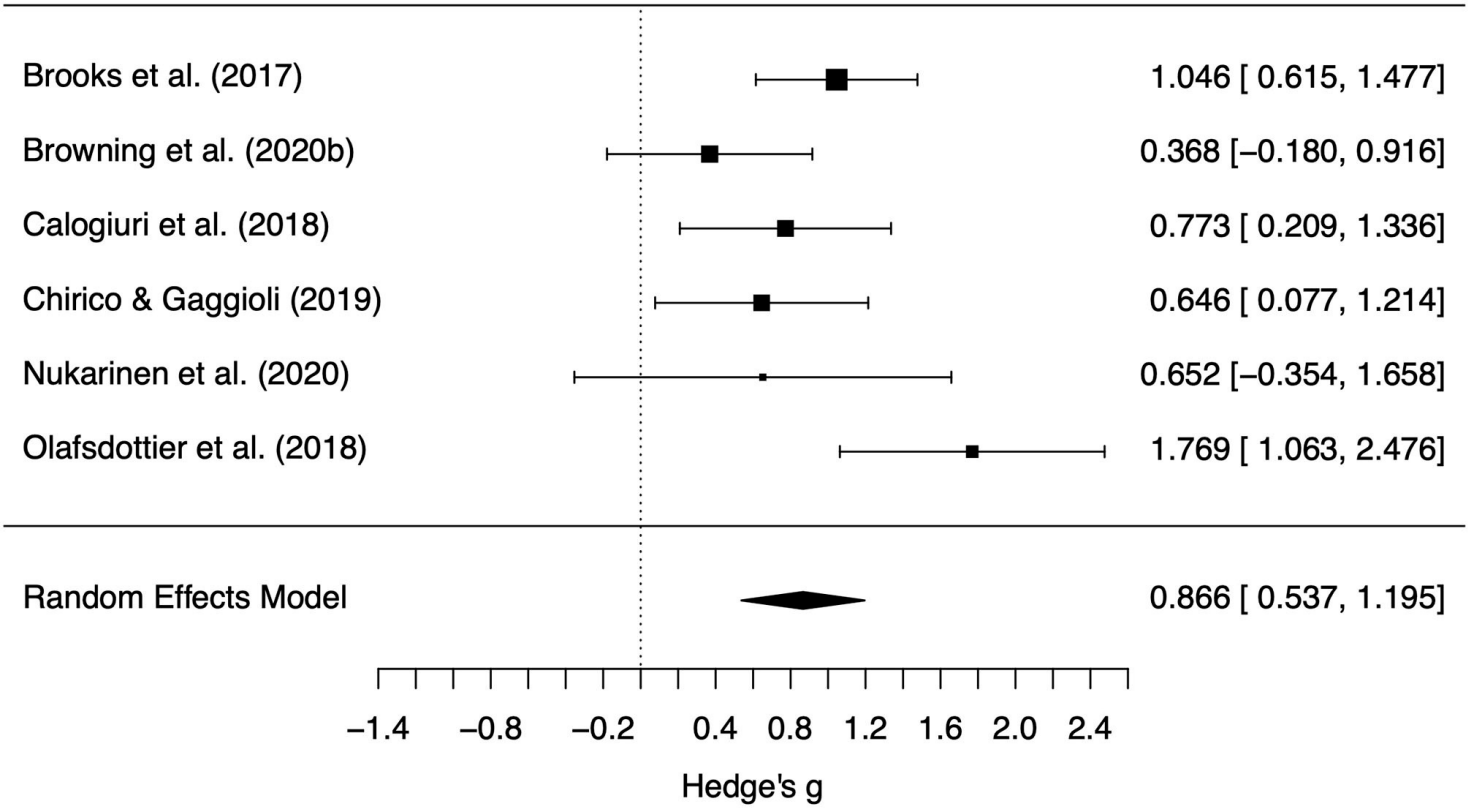
Forest plot of standardized mean difference positive affect change scores between the actual and simulated natural settings. Points indicate the estimated effect for each study with variance estimates indicated by the size of the point and width of the error bars. Scores above 0 indicate greater increase in positive affect in the actual natural setting.

**Figure 4:**
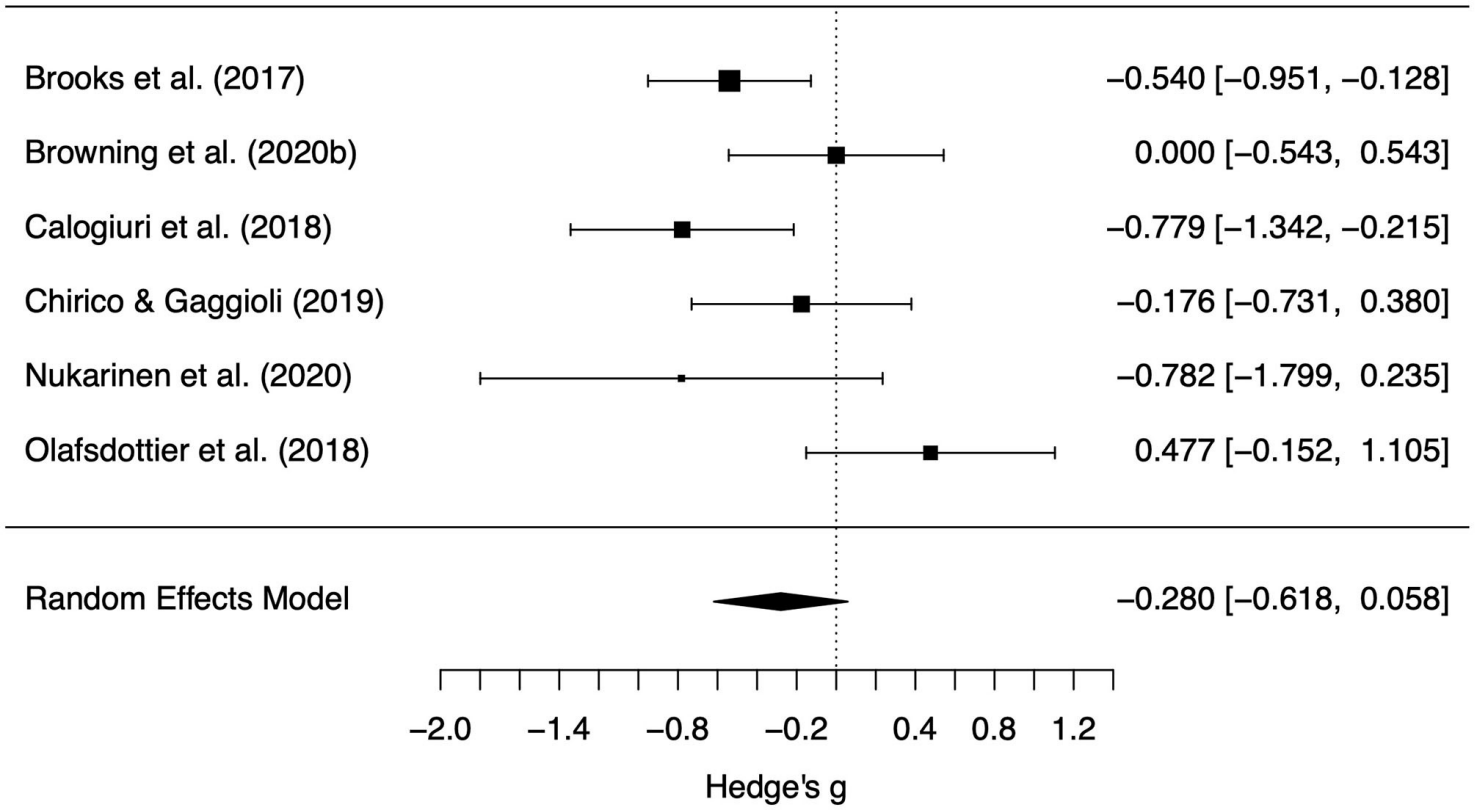
Forest plot of standardized mean difference negative affect change scores between the actual and simulated natural settings. Points indicate the estimated effect for each study with variance estimates indicated by the size of the point and width of the error bars. Scores below 0 indicate greater reduction in negative affect in the actual natural setting.

We found a large difference between the positive affect change scores for the different settings (*g* = 0.87, *z* = 5.16, *p* < 0.001, 95% CI = 0.54, 1.20, see Figure 3). More specifically, the actual setting promoted beneficial changes in positive affect much more than the simulated setting. Little difference between setting types was observed for the negative affect change scores (*g* = −0.28, *z* = –1.62, *p* = 0.10, 95% CI = −0.62, 0.06, see Figure 4). The differences in change scores for the simulated and actual settings are provided for each experiment in Table S3. One can see there that, for positive affect, a difference in change scores typically shows increases from actual settings and decreases from simulated settings; that is, it appears that the simulated settings tended to reduce feeling attentive, active, alert, excited, enthusiastic, determined, inspired, proud, interested, and/or strong, while actual settings had the opposite effect. In contrast, both settings tended to show decreases in negative affect including feeling afraid, ashamed, distressed, guilty, hostile, irritable, jittery, nervous, scared, and/or upset. Removal of any single study did not change the conclusions; differences in effects between actual and natural settings for positive affect remained statistically significant and effect sizes remained large, and differences in effects for negative affect remained marginal/non-significant (see Table S4). Change scores showed moderate heterogeneity for negative affect [Q(5) = 12.2, *p* = 0.033, *I*^2^ = 50.7%, T2 = 0.09] and positive affect [Q(5) = 11.0, *p* = 0.052, *I*^2^ = 44.7%, T2 = 0.07].

### Publication Bias

Doi plots showed symmetric spread of effect sizes against Z scores, suggesting no substantive publication bias (see Figure S1). This conclusion was supported by the LFK index of 0.55 for positive affect and −1.23 for negative affect.

### Quality of Evidence

Nearly all studies suffered from potential biases; none reported blinding participants/personnel to conditions and blinding participants to outcome assessments. However, when viewed more holistically, two studies showed low risk of bias across the majority of bias domains (see Figure S2). The remaining four studies showed unclear/high risk of bias in the majority of bias domains.

The evidence for more beneficial change in positive affect for actual vs. simulated natural settings was *Moderate*. Positive affect results showed high consistency and strength of associations; however, there were too few studies and too much heterogeneity to classify the evidence as *Robust*. In contrast, the evidence for differences in negative affect was deemed to be *Slight* due to low consistency and strength of associations.

## Discussion

### Summary and Interpretation of Main Findings

Simulations of natural settings are increasingly used for health promotion in scenarios where physical exposure is not possible (White et al., 2018). Several years ago, McMahan and Estes (2015) found indirect evidence that the effects of simulated natural settings on mood were smaller than the effects of actual natural settings on mood. The current meta-analysis extends their work by limiting our assessment to studies that directly compared the same (or very similar) settings. We identified 16 studies that have examined this topic but only 6 that have used a cross-cutting, standardized measure of mood before and after exposure. Pooled change scores showed a large difference between the effects of actual vs. simulated settings on positive affect. There was little difference between settings for negative affect. Although more research is needed in this emerging line of research, the available data indicate that going outdoors into natural settings is likely better at supporting mood than remaining indoors in simulated natural settings.

Our central finding—that actual natural settings benefit mood more than simulated natural settings—reflect on the different potential of the two settings to activate pathways that with repeated exposures can cumulatively benefit health. A person who goes outdoors into an actual natural setting can potentially activate pathways to health in three domains: reducing exposure to harmful anthropogenic features of the environment (mitigation), building capacities (instoration), and renewing depleted capacities (restoration) (see Figure 1) (Markevych et al., 2017). Enhanced mood may be an active component of a pathway or a concomitant of its operation. The mitigation domain encompasses pathways by which vegetation and other features of a natural setting offer protection from air pollution, noise, heat, visual blight, privacy intrusions, and other harmful features of urban environments in which they might otherwise spend time, during leisure or otherwise (Gopalakrishnan et al., 2019). The instoration domain includes pathways in which natural settings serve as a context for health-promoting behaviors, such as physical activity, social interaction, and exposure to microbial diversity (Dobetsberger and Buchbauer, 2011; Rook et al., 2014; Łaszewska et al., 2018), which in turn modify neurochemical pathways in the gut and brain that appear to stabilize mood (Clarke et al., 2013). The restoration domain includes pathways by which nature experience can promote the renewal of depleted adaptive resources, as through stress recovery and directed attention restoration (Ulrich, 1983; Kaplan, 1995). In the context of nature experience in an actual outdoor setting, pathways in any one domain may become engaged to a greater degree, as when sensory richness and opportunities for exploration sustain engagement with the environment and so a restorative process. In addition, pathways in all three domains may work in mutually reinforcing ways that cannot get realized with simulations (e.g., as when neighbors enjoy their social contact and fresh air when walking together in a nearby park to wind down after a difficult day at work) (Hartig et al., 2014). For these reasons, the benefits of an actual natural setting can be expected to extend beyond the benefits available when only pathways associated with auditory and visual sensory inputs get activated to a lesser degree by simulation technologies (Horiuchi et al., 2014).

The studies we identified in our literature review but excluded from the meta-analysis showed similar findings as our pooled effects—at least for affective valence—which reinforces confidence in our conclusions. Three studies that compared actual nature with its virtual counterpart but were excluded for various reasons (see Methods) also showed stronger mood effects for actual nature than for simulated nature (Hartig et al., 1997; Mayer et al., 2009; Ryan et al., 2010). Findings from other studies that examined differences in affective arousal (i.e., energy and vigor) between actual and simulated natural settings were mixed. One found stronger beneficial effects for actual nature than for simulated nature (Kjellgren and Buhrkall, 2010), but two others showed similar effects between these two types of exposures (Plante et al., 2006; Yin et al., 2018). Collectively, these excluded studies point to our findings with PANAS extending to other measures of mood.

These findings provide evidence for public health messaging that encourages people to go outdoors into natural settings rather than stay indoors, even if simulations of natural settings are utilized. There are other important outcomes of encouraging people to visit actual natural settings of course. Living vegetation and other features provide myriad ecosystem services beyond the cultural domain that encompasses human health and well-being, such as provisioning of food and clean water (Bratman et al., 2019; Keeler et al., 2019), which can be better realized by local residents if a connection with these settings is built over repeated visitation (Richardson et al., 2016; Colléony et al., 2019; Rosa and Collado, 2019). Finally, ethical sensibilities could encourage the protection of the possibility for other forms of life to develop and thrive, entirely aside from their utility to humans (Leopold, 1949; Hartig, 1993).

However, access to actual natural settings is often not available for shorter and longer periods to many who could benefit from it. Should simulations then be offered as an alternative going outdoors into natural settings? The results we report here encourage caution in this regard; they show that positive affect declined while viewing most of the simulations. This stands in contrast to much other research and encourages questions about differences between the simulations and other methods of the experiments studied here and those used in experiments that found beneficial outcomes. These matters need focused research attention, as the potential for therapeutic applications is great (White et al., 2018). Nevertheless, in contexts such as hospitals and prisons and with social distancing as during the COVID-19 pandemic, simulations may offer the only options for experiencing nature. Indeed, simulations may be safer therapeutic modalities than going outdoors and risking allergies, infectious disease, and accidental injury (Jennings et al., 2019). Simulations also provide the clinician with greater control than they would have with other nature-based therapies, such as forest bathing and park prescriptions, which are challenged by low levels of patience adherence and high levels of heterogeneity regarding the “treatment” patients receive (Kamioka et al., 2012; Crnic and Kondo, 2019). Lastly, simulations are practical; they can be safely and quickly moved from one person or group to another or shared at little/no cost through online streaming. Specific contexts where simulated natural settings may be particularly valuable were recently reviewed by Litleskare et al. (2020) and include palliative treatment in clinical settings, stress management in the workplace, mental health and cognitive development in school settings, and nature experiences for space missions. Personnel in other confined situations such as those found in submarines, Arctic and Antarctic polar bases, and medical imaging equipment like MRIs or CAT scans might also benefit from simulations (Anderson et al., 2017).

### Strengths, Limitations, and Future Research Recommendations

The modest number of included studies meant limited representation of natural settings. It also limited our statistical power. Our marginal result for negative affect could have resulted from the high levels of between-study variation. To overcome the low power of classic publication bias tests, we employed a novel method heralded in recent years as a more powerful alternative (Furuya-Kanamori et al., 2018). However, with just six studies, power for these tests was still on the low end; thus, there could still have been publication bias in the studies identified above. Additional studies that directly compare mood effects between actual and simulated nature would provide more robust meta-analytic findings as a result of lower levels of heterogeneity.

The sample size was in part a result of our least common denominator research design approach. We included studies with only pre- and post-condition measures of PANAS. Like all studies, meta-analyses require a degree of researcher decision-making that can influence the results. We chose our inclusion/exclusion criteria because, based on our critical review, it allowed the greatest number of effects from experimental studies to pool together. It is worthwhile to investigate whether the employment of meta-analytic approaches could result in pooled effects that diverge from what a larger (less restricted) body of literature generally shows. Therefore, there is value in examination by other researchers of the effects of actual vs. simulated nature not only on other dimensions of mood but also on human health/well-being more generally.

The circumstances under which simulations can reliably engender desired beneficial outcomes warrant further research. Needed studies would address not only the features of the simulations (e.g., sampling of environmental features, quality of representation of the actual environment, and degree of immersion) and the features of the context in which they get presented (e.g., activity and duration) but also the circumstances and needs of those who would view them vs. entering an actual setting. For example, none of the experiments we reviewed had a stress or mental fatigue induction prior to the environmental treatment. This lack of a need to renew depleted resources may have led those participants to dislike their simulation experience rather than enjoy it as a restorative respite. Similarly, some populations, such as prisoners, may find that simulations only remind them of constraints they cannot escape; they may resent the simulations rather than appreciate them (Moran, 2019).

Further research on the relative benefits of simulated and actual nature should also employ stronger study designs. When possible, blinding to comparisons may help. Actual exposure as studied here generally requires that people travel to a natural setting. These pretreatment exposures could have initiated activation of pathways that primed participants to respond to natural settings differently. Such effects would have been difficult to replicate for the simulation conditions without also providing the participant with exposure to actual settings, thereby combining portions of exposure types. One method that was developed by Chirico and Gaggioli (2019) and that helps overcome this potential bias is to bring all participants to an outdoor location and then ask them to take part in their assigned condition: donning a head-mounted display or focusing on the actual setting before them, for example. Of course, participants ultimately understood that they were watching a simulation—not observing the actual landscape outdoors—when the headset was turned on. Reducing bias, therefore, may be only partially solved in studies that compare simulated and actual natural settings through between-subjects experimental design in which participants are blinded to the conditions other participants are assigned.

Our study also had several strengths; most notably, our meta-analytic approach allowed us to calculate the effect size describing mood changes in ways that other approaches (i.e., narrative and systematic reviews) would not have been able to do. In addition, the chosen outcomes—negative and positive affect—are sensitive to the operation of multiple pathways by which nature exposures and experiences can influence health, and they showed more consistent effects following short-term exposure to physical natural settings than other intermediate psychological or physiological outcomes that cumulatively over time affect health in lasting ways (McMahan and Estes, 2015; Kondo et al., 2018). Moreover, positive change in mood is a prevalent outcome of diverse leisure activities, valuable in its own right and for the persistent influence it exerts on postleisure behavioral processes of relevance to adaptive functioning and health (Hull, 2018). Presumably, then, just as mood levels change more strongly in actual nature, diverse other outcomes are likely better realized by going outdoors.

## Conclusion

In closing, we recognize the promise of simulation technology and currently participate in its further development, for example as a means to represent alternative future environments that would result from different planning choices (Lindal and Hartig, 2013, 2015; Joseph et al., 2020). However, we think that decision-makers and the publics they serve should appreciate the limits of simulations identified here and avoid assuming they can simply substitute for the real thing.

## Data Availability Statement

All datasets generated for this study are included in the article/Supplementary Material.

## Author Contributions

MB: conceptualization, methodology, investigation, resources, data curation, writing, visualization, supervision, and project administration. NS: methodology, software, validation, formal analysis, investigation, data curation, writing, and visualization. TH: critical review and writing. DB: software, data curation, and writing. C-PY: critical review and writing, OM: data curation and writing. AD: methodology, software, validation, formal analysis, investigation, and writing. All authors contributed to the article and approved the submitted version.

## Conflict of Interest

The authors declare that the research was conducted in the absence of any commercial or financial relationships that could be construed as a potential conflict of interest.

## Footnotes

^1^A systematic review on all health effects associated with outdoor “green” exercise versus indoor exercise with simulations of natural settings was published after the current study began (Lahart et al., 2019).

## References

[B1] Anderson A. P. Mayer M. D. Fellows A. M. Cowan D. R. Hegel M. T. Buckey J. C. (2017). Relaxation with immersive natural scenes presented using virtual reality. Aerospace Medicine Human Perform. 88, 520–526. 10.3357/AMHP.4747.2017 28539139

[B2] Balshem H. Helfand M. Schünemann H. J. Oxman A. D. Kunz R. Brozek J.. (2011). GRADE guidelines: 3. Rating the quality of evidence. J. Clin. Epidemiol. 64, 401–406. 10.1016/j.jclinepi.2010.07.015 21208779

[B3] Belur J. Tompson L. Thornton A. Simon M. (2018). Interrater reliability in systematic review methodology: exploring variation in coder decision-making. Sociol. Methods Res. 13:9372 10.1177/0049124118799372

[B4] Beyer K. Szabo A. Hoormann K. Stolley M. (2018). Time spent outdoors, activity levels, and chronic disease among American adults. J. Behav. Med. 41, 494–503. 10.1007/s10865-018-9911-1 29383535 PMC6031452

[B5] Birenboim A. Dijst M. Ettema D. de Kruijf J. de Leeuw G. Dogterom N. (2019). The utilization of immersive virtual environments for the investigation of environmental preferences. Landsc. Urban Plan 189, 129–138. 10.1016/j.landurbplan.2019.04.011

[B6] Bratman G. N. Anderson C. B. Berman M. G. Cochran B. de Vries S. Flanders J.. (2019). Nature and mental health: an ecosystem service perspective. Sci. Adv. 5:eaax0903. 10.1126/sciadv.aax0903 31355340 PMC6656547

[B7] Brooks A. M. Ottley K. M. Arbuthnott K. D. Sevigny P. (2017). Nature-related mood effects: Season and type of nature contact. J. Environ. Psych. 54, 91–102. 10.1016/j.jenvp.2017.10.004

[B8] Browning M. Mimnaugh K. J. van Riper C. J. Laurent H. K. LaValle S. M. (2020b). Can simulated nature support mental health? Comparing short, single-doses of 360-degree nature videos in virtual reality with the outdoors. Front. Psychol. 10.3389/fpsyg.2020.02200 32010003 PMC6974516

[B9] Browning M. Saeidi-Rizi F. McAnirlin O. Yoon H. Pei Y. (2020a). The role of methodological choices in the effects of experimental exposure to simulated natural landscapes on human health and cognitive performance: a systematic review. Environ. Behav. 7, 1–43. 10.1177/0013916520906481

[B10] Browning M. Suppakittpaisarn P. Jiang S. Joseph A. (2020c). Human health assessments of green infrastructure designs using virtual reality. Landsc. Archit. 27 10.14085/j.fjyl.2020.09.0000.15

[B11] Burrows B. Browning M. Solai K. Fast D. Litbarg N. O. Moskowitz J. T. (2020). Fully immersive virtual reality-based mindfulness intervention in hemodialysis patients: a pilot study assessing safety and utility, in Proceedings from the 2020 American Society of Nephrology Kidney; 2020, Fully Online Meeting.

[B12] Calogiuri G. Litleskare S. Fagerheim K. A. Rydgren T. L. Brambilla E. Thurston M. (2018). Experiencing nature through immersive virtual environments: environmental perceptions, physical engagement, and affective responses during a simulated nature walk. Front. Psychol. 8:8661. 10.3389/fpsyg.2017.02321 29410635 PMC5787081

[B13] Card N. A. (2015). Applied Meta-Analysis for Social Science Research. New York, NY: Guilford Publications.

[B14] Chirico A. Gaggioli A. (2019). When virtual feels real: comparing emotional responses and presence in virtual and natural environments. Cyberpsychol. Behav. Soc. Netw. 22, 220–226. 10.1089/cyber.2018.0393 30730222

[B15] Clarke G. Grenham S. Scully P. Fitzgerald P. Moloney R. D. Shanahan F.. (2013). The microbiome-gut-brain axis during early life regulates the hippocampal serotonergic system in a sex-dependent manner. Mol. Psychiatry 18, 666–673. 10.1038/mp.2012.77 22688187

[B16] Colléony A. White R. Shwartz A. (2019). The influence of spending time outside on experience of nature and environmental attitudes. Landsc. Urban Plan 187, 96–104. 10.1016/j.landurbplan.2019.03.010

[B17] Coste J. Guillemin F. Pouchot J. Fermanian J. (1997). Methodological approaches to shortening composite measurement scales. J. Clin. Psychol. 50, 247–252. 10.1016/S0895-4356(96)00363-0 9120523

[B18] Crnic M. Kondo M. C. (2019). Nature rx: Reemergence of pediatric nature-based therapeutic programs from the late 19th and early 20th centuries. Am. J. Public Health 109, 1371–1378. 10.2105/AJPH.2019.305204 31415211 PMC6727277

[B19] Davis S. Nesbitt K. Nalivaiko E. (2014). A systematic review of cybersickness, in Proceedings from the 2014 Conference on Interactive Entertainment; 2014 Dec, New York, New York, USA 1–9.

[B20] Dickersin K. Scherer R. Lefebvre C. (1994). Identifying relevant studies for systematic reviews. BMJ 309:1286. 10.1136/bmj.309.6964.1286 7718048 PMC2541778

[B21] Dobetsberger C. Buchbauer G. (2011). Actions of essential oils on the central nervous system: an updated review. Flavour Fragr. J. 26, 300–316. 10.1002/ffj.2045

[B22] Doi S. A. R. Furuya-Kanamori L. Thalib L. Barendregt J. J. (2017). Meta-analysis in evidence-based healthcare: A paradigm shift away from random effects is overdue. Int. J. Evid. Based Healthc. 15, 152–160. 10.1097/XEB.0000000000000125 29135532

[B23] Dzhambov A. M. Lercher P. (2019). Road traffic noise exposure and birth outcomes: an updated systematic review and meta-analysis. IJERPH 16, 2522–20. 10.3390/ijerph16142522 31311086 PMC6678260

[B24] Frankenhaeuser M. Johansson G. (1986). Stress at work: psychobiological and psychosocial aspects. Appl. Psychol. 35, 287–299. 10.1111/j.1464-0597.1986.tb00928.x

[B25] Furuya-Kanamori L. Barendregt J. J. Doi S. A. R. (2018). A new improved graphical and quantitative method for detecting bias in meta-analysis. Int. J. Evid. Based Healthc 16, 195–203. 10.1097/XEB.0000000000000141 29621038

[B26] Furuya-Kanamori L. Xu C. Lin L. Doan T. Chu H. Thalib L.. (2020). P value-driven methods were underpowered to detect publication bias: analysis of Cochrane review meta-analyses. J. Clin. Epidemiol. 118, 86–92. 10.1016/j.jclinepi.2019.11.011 31743750

[B27] Gatersleben B. Andrews M. (2013). When walking in nature is not restorative-The role of prospect and refuge. Health Place 20, 91–101. 10.1016/j.healthplace.2013.01.001 23399852

[B28] Gopalakrishnan V. Ziv G. Bakshi B. R. (2019). Role of vegetation in mitigating air emissions across industrial sites in the US. ACS Sustainable Chem. Eng. 7, 3783–3791. 10.1021/acssuschemeng.8b04360

[B29] Hartig T. (1993). Nature experience in transactional perspective. Landsc. Urban Plan 25, 17–36. 10.1016/0169-2046(93)90120-3

[B30] Hartig T. Astell-Burt T. Bergsten Z. Amcoff J. Mitchell R. J. Feng X. (2020). Associations between greenspace and mortality vary across contexts of community change: a longitudinal ecological study. J. Epi. Comm. Health 74, 1–7. 10.1136/jech-2019-213443 32132229 PMC7320793

[B31] Hartig T. Korpela K. M. Evans G. W. Gärling T. (1997). A measure of restorative quality in environments. Scandinavian Housing Planning Res. 14, 175–194. 10.1080/02815739708730435

[B32] Hartig T. Mitchell R. J. de Vries S. Frumkin H. (2014). Nature and health. Ann. Rev. Public Health 35, 207–228. 10.1146/annurev-publhealth-032013-182443 24387090

[B33] Haydock D. Moran A. (2019). Modern Indoor Living can be Bad for Your Health: New YouGov Survey Sheds Light on Risk of the “Indoor Generation.” *www.velux.com*, 1–2. Available online at: https://www.veluxusa.com/media/press-kits/indoor-generation (accessed September 10 2019).

[B34] Hedges L. V. Olkin I. (2014). Statistical Methods for Meta-Analysis. Orlando, FL: Academic Press.

[B35] Higgins J. P. T. Altman D. G. Gøtzsche P. C. Jüni P. Moher D. Oxman A. D.. (2011). The Cochrane Collaboration's tool for assessing risk of bias in randomised trials. BMJ 343:d5928. 10.1136/bmj.d5928 22008217 PMC3196245

[B36] Higgins J. P. T. Green S. (2011a). Identifying and measuring heterogeneity, in Cochrane Handbook for Systematic Reviews of Interventions, eds Higgins J. P. T. Green S. (The Cochrane Collaboration).

[B37] Higgins J. P. T. Green S. (2011b). Imputing standard deviations for changes from baseline, in Cochrane Handbook for Systematic Reviews of Interventions, eds Higgins J. P. T. Green S. (The Cochrane Collaboration), 1–3.

[B38] Horiuchi M. Endo J. Takayama N. Murase K. Nishiyama N. Saito H.. (2014). Impact of viewing vs. not viewing a real forest on physiological and psychological responses in the same setting. IJERPH 11, 10883–10901. 10.3390/ijerph111010883 25333924 PMC4211012

[B39] Howard M. C. (2017). A meta-analysis and systematic literature review of virtual reality rehabilitation programs. Comput. Human Behav. 70, 317–327. 10.1016/j.chb.2017.01.013

[B40] Huang S.-C. L. (2009). The validity of visual surrogates for representing waterscapes. Landscape Res. 34, 323–335. 10.1080/01426390902867984

[B41] Hull R. B. IV. (2018). Mood as a product of leisure: causes and consequences. JLR 22, 99–111. 10.1080/00222216.1990.11969818

[B42] Jennings V. L. Browning M. Rigolon A. (2019). Friend or foe? An overview of the services and disservices from urban green spaces, in Urban Green Spaces Public Health and Sustainability in the United States, eds Jennings V. L. Browning M. Rigolon A. (Cham: Springer International Publishing), 7–30.

[B43] Joseph A. Browning M. Jiang S. (2020). Using immersive virtual environments (IVEs) to conduct environmental design research: A primer and decision framework. HERD 13, 11–25. 10.1177/1937586720924787 32436436

[B44] Kahn P. H. Jr. Friedman B. Gill B. Hagman J. Severson R. L. (2008). A plasma display window?—The shifting baseline problem in a technologically mediated natural world. J. Environ. Psych. 28, 192–199. 10.1016/j.jenvp.2007.10.008

[B45] Kamioka H. Tsutani K. Mutoh Y. Honda T. Shiozawa N. Park S.. (2012). A systematic review of randomized controlled trials on curative and health enhancement effects of forest therapy. PRBM. 5, 85–11. 10.2147/PRBM.S32402 22888281 PMC3414249

[B46] Kaplan S. (1995). The restorative benefits of nature: Toward an integrative framework. J. Environ. Psych. 15, 169–182. 10.1016/0272-4944(95)90001-2

[B47] Keeler B. L. Hamel P. McPhearson T. Hamann M. H. Donahue M. L. Prado K. A. M. (2019). Social-ecological and technological factors moderate the value of urban nature. Nature Sustainability 2, 1–10. 10.1038/s41893-018-0202-1

[B48] Kensinger E. A. Corkin S. (2004). Two routes to emotional memory: distinct neural processes for valence and arousal. Proc. Natl. Acad. Sci. U.S.A. 101, 3310–3315. 10.1073/pnas.0306408101 14981255 PMC365786

[B49] Kjellgren A. Buhrkall H. (2010). A comparison of the restorative effect of a natural environment with that of a simulated natural environment. J. Environ. Psych. 30, 464–472. 10.1016/j.jenvp.2010.01.011

[B50] Kondo M. C. Fluehr J. McKeon T. Branas C. C. (2018). Urban green space and its impact on human health. IJERPH 15, 1–28. 10.3390/ijerph15030445 29510520 PMC5876990

[B51] Lahart I. Darcy P. Gidlow C. J. Calogiuri G. (2019). The effects of green exercise on physical and mental wellbeing: a systematic review. IJERPH 16, 1352–26. 10.3390/ijerph16081352 30991724 PMC6518264

[B52] Lassonde K. A. Gloth C. A. Borchert K. (2012). Windowless classrooms or a virtual window world. Teaching Psychol. 39, 262–267. 10.1177/0098628312456618

[B53] Łaszewska K. Goroncy A. Weber P. Pracki T. Tafil-Klawe M. (2018). Influence of the spectral quality of light on daytime alertness levels in humans. Adv. Cogn. Psychol. 14, 192–208. 10.5709/acp-0250-0 32509040 PMC7263078

[B54] LaValle S. M. (2017). Virtual Reality. Cambridge, UK: Cambridge University Press.

[B55] LaViola J. J. Jr. (2000). A discussion of cybersickness in virtual environments. SIGCHI Bull. 32, 47–56. 10.1145/333329.333344

[B56] Leopold A. (1949). A Sand County Almanac. New York, NY: Oxford University Press.

[B57] Lindal P. J. Hartig T. (2013). Architectural variation, building height, and the restorative quality of urban residential streetscapes. J. Environ. Psych. 33, 26–36. 10.1016/j.jenvp.2012.09.003

[B58] Lindal P. J. Hartig T. (2015). Effects of urban street vegetation on judgments of restoration likelihood. Urban Urban Gree 14, 200–209. 10.1016/j.ufug.2015.02.001

[B59] Litleskare S. E. MacIntyre T. Calogiuri G. (2020). Enable, reconnect and augment: a new era of virtual nature research and application. IJERPH 17, 1–19. 10.3390/ijerph17051738 32155911 PMC7084893

[B60] Markevych I. Schoierer J. Hartig T. Chudnovsky A. Hystad P. Dzhambov A. M.. (2017). Exploring pathways linking greenspace to health: Theoretical and methodological guidance. Environ. Res. 158, 301–317. 10.1016/j.envres.2017.06.028 28672128

[B61] Mayer F. S. Frantz C. M. Bruehlman-Senecal E. Dolliver K. (2009). Why is nature beneficial?: the role of connectedness to nature. Environ. Behav. 41, 607–643. 10.1177/0013916508319745

[B62] McMahan E. A. Estes D. (2015). The effect of contact with natural environments on positive and negative affect: a meta-analysis. J. Posit. Psychol. 10, 507–519. 10.1080/17439760.2014.994224

[B63] Moher D. Liberati A. Tetzlaff J. Altman D. G. The PRISMA Group (2009). Preferred reporting items for systematic reviews and meta-analyses: The PRISMA statement. Ann. Intern. Med. 151, 264–9–W64. 10.7326/0003-4819-151-4-200908180-00135 19622511

[B64] Moran D. (2019). Back to nature? Attention restoration theory and the restorative effects of nature contact in prison. Health Place 57, 35–43. 10.1016/j.healthplace.2019.03.005 30953997

[B65] Nukarinen T. Istance H. O. Rantala J. Mäkel,ä J. Korpela K. M. Ronkainen K. (2020). Physiological and Psychological Restoration in Matched Real and Virtual Natural Environments eds Bernhaupt R. Mueller F. Verweij D. Andres J. McGrenere J. Cockburn A. (Honolulu, HI), 1–8. 10.1145/3334480.3382956

[B66] Olafsdottir G. Cloke P. Schulz A. van Dyck Z. Eysteinsson T. Thorleifsdottir B. (2018). Health benefits of walking in nature: a randomized controlled study under conditions of real-life stress. Environ. Behav. 23, 1–27.

[B67] Plante T. G. Cage C. Clements S. Stover A. (2006). Psychological benefits of exercise paired with virtual reality: Outdoor exercise energizes whereas indoor virtual exercise relaxes. Int. J. Stress Manag. 13, 108–117. 10.1037/1072-5245.13.1.108

[B68] Radke E. G. Braun J. M. Nachman R. M. Cooper G. S. (2020). Phthalate exposure and neurodevelopment-A systematic review and meta-analysis of human epidemiological evidence. Environ. Int. 137:105408. 10.1016/j.envint.2019.105408 32045779 PMC8453372

[B69] Richardson M. Cormack A. McRobert L. Underhill R. (2016). 30 Days wild: Development and evaluation of a large-scale nature engagement campaign to improve well-being. PLoS ONE 11:e149777. 10.1371/journal.pone.0149777 26890891 PMC4758721

[B70] Riva G. Bernardelli L. Browning M. Castelnuovo G. Cavedoni S. Chirico A. (2020). COVID feel good - an easy self-help virtual reality protocol to overcome the psychological burden of Coronavirus. Front. Psychiatry 10.3389/fpsyt.2020.563319 PMC7538634 33173511

[B71] Rook G. A. Raison C. L. Lowry C. A. (2014). Microbial “old friends,” immunoregulation and socioeconomic status. Clin. Exp. Immunol. 177, 1–12. 10.1111/cei.12269 24401109 PMC4089149

[B72] Rosa C. D. Collado S. (2019). Experiences in nature and environmental attitudes and behaviors: Setting the ground for future research. Front. Psychol. 10:763. 10.3389/fpsyg.2019.00763 31024400 PMC6465548

[B73] Rosenthal R. (2009). Parametric measures of effect size, in The Handbook of Research Synthesis and Meta-Analysis, eds Cooper H. Hedges L. V. (New York, NY: Russell Sage Foundation), 231–244.

[B74] Rossetti T. Hurtubia R. (2020). An assessment of the ecological validity of immersive videos in stated preference surveys. J. Choice Modell. 34, 1–12. 10.1016/j.jocm.2019.100198

[B75] Ryan R. M. Weinstein N. Bernstein J. Brown K. W. Mistretta L. Gagne M. (2010). Vitalizing effects of being outdoors and in nature. J. Environ. Psych. 30, 159–168. 10.1016/j.jenvp.2009.10.009

[B76] Twohig-Bennett C. Jones A. (2018). The health benefits of the great outdoors: a systematic review and meta-analysis of greenspace exposure and health outcomes. Environ. Res. 166, 628–637. 10.1016/j.envres.2018.06.030 29982151 PMC6562165

[B77] Ulrich R. S. (1983). Aesthetic and affective response to natural environment, in Behavior and the Natural Environment, eds Altman I. Wohlwill J. F. (Boston, MA: Springer), 85–125.

[B78] Viechtbauer W. (2010). Conducting meta-analyses in R with the metafor package. J. Stat. Softw. 36, 1–48. 10.18637/jss.v036.i03

[B79] White M. P. Yeo N. Vassiljev P. Lundstedt R. Wallergård M. Albin M.. (2018). A prescription for “nature” - The potential of using virtual nature in therapeutics. Neuropsychiatr. Dis. Treat. 14, 3001–3013. 10.2147/NDT.S179038 30510422 PMC6231506

[B80] Yin J. Zhu S. MacNaughton P. Allen J. G. Spengler J. D. (2018). Physiological and cognitive performance of exposure to biophilic indoor environment. Build. Environ. 132, 255–262. 10.1016/j.buildenv.2018.01.006

