# Supplementary material for: An Actual Natural Setting Improves Mood Better Than Its Virtual Counterpart: A Meta-Analysis of Experimental Data
Source: Front Psychol. 2020 Sep 30;11:2200. doi: 10.3389/fpsyg.2020.02200 (PMC7554239; doi:10.3389/fpsyg.2020.02200)
Supplement: Supplementary file 1 [file Data_Sheet_1.docx]

**Supplementary Materials**

An actual natural setting improves mood better than its virtual counterpart: A meta-analysis of experimental data

*Matthew H. E. M. Browning*, Nathan Shipley, Terry Hartig, Chia-Pin Yu, Douglas Becker, Olivia McAnirlin & Angel M. Dzhambov*

^*^mhb2@clemson.edu, 263 Lehotsky Hall, Clemson, SC 29634-0735, USA

| **Table S1.** Search terms that were used to identify articles in Browning et al. (2020) and that were later screened for consideration in their review and the current meta-analysis. |
| --- |
| **Natural landscape** |
| 1. "Natural environment*"  2. “Nature environment*”  3. “Natural and urban environment*”  4. “Natural and built environment*”  4. "Restorative environment*" 5. "Natural landscape*"  6. “Natural and urban landscape*”  7. “Natural and built landscape*”  7. "Nature scene*"  8. “Natural scene*”  9. “Natural and urban scene*”  10. “Natural and built scene*”  10. “Natural setting*”  11. “Natural and urban setting*”  12. “Natural and built setting*”  12. “Pastoral environment*”  13. “Pastoral scene*”  14. “Pastoral setting*”  15. “Pastoral landscape*” 15. "Nearby nature"  16. “Natural space*”  17. “Nature view*”  18. “Natural view*”  19. “Green space*”  20. “Greenspace*”  21. “Blue space*”  22. “Bluespace*”  23. “Natural versus built environment*”  24. “Natural versus urban environment*”  25. “Natural versus built landscape*”  26. “Natural versus urban landscape*” 27. 1 OR 2 OR 3 OR 4 OR 5 OR 6 OR 7 OR 8 OR 9 OR 10 OR 11 OR 12 OR 13 OR 14 OR 15 OR 16 OR 17 OR 18 OR 21 OR 22 OR 23 OR 24 OR 25 OR 26 |

| **Table S2.** Descriptions of domains used to evaluate methodological biases in reviewed studies. | |
| --- | --- |
| **Domain** | **Description** |
| 1. Random sequence generation | Whether the method used to generate the allocation sequence was described in sufficient detail to allow assessment of whether it should produce comparable groups |
| 2. Allocation concealment | Whether the method used to conceal the allocation sequence was described in sufficient detail to determine whether intervention allocations could have been foreseen |
| 3. Blinding of participants and personnel | Whether adequate measures were used to limit the extent to which participants and researchers could realize the condition to which they were assigned/administering and know the full suite of conditions that were assigned |
| 4. Blinding of outcome assessment | Whether adequate measures were used to blind outcome assessment from knowledge of which intervention a participant received |
| 5. Incomplete outcome data | Whether accounting of data was complete for each main outcome, including attrition and exclusions from the analysis |
| 6. Reporting bias | Whether outcomes were selectively reported |
| 7. Other biases | Whether any important concerns not covered in other domains were present |

**Table S3.** Summary of mean change scores in mood resulting from exposure to actual versus simulated natural settings.

|  | **Positive affect change score** | | | **Negative affect change score** | | |
| --- | --- | --- | --- | --- | --- | --- |
| Study | Actual | Simulated | Actual vs. simulated | Actual | Simulated | Actual vs. simulated |
| Brooks et al. (2017) | **0.3** | **-0.3** | 0.6 | **-0.2** | 0.0 | -0.2 |
| Browning et al. (2020a) | **0.18** | **0.01** | 0.17 | **-0.07** | **-0.07** | 0.0 |
| Calogiuri et al. (2018) | **0.02** | **-0.08** | 0.1 | **-0.14** | **0.29** | -0.43 |
| Chirico and Gaggioli (2019) | **0.32** | **-0.08** | 0.4 | **-0.41** | **-0.31** | -0.1 |
| Nukarinen et al. (2020) | **0.5** | **-3.38** | 3.87 | **-4.38** | **-2.5** | -1.88 |
| Olafsdottir et al. (2018) | **5.7** | **-6.0** | 11.7 | **-1.94** | **-3.22** | 1.28 |

*Beneficial effects* for positive affect (positive values) and negative affect (negative values) are **shown in bold**.

*Harmful effects* for positive affect (negative values) and negative affect (positive values) are **shown in bold and are underlined**.

**Table S4**. Pooled effect estimates for meta-analyses of positive affect change scores after excluding each study individually. The excluded study is indicated in the first column of each row.

| **Study** | **Estimate** | **SE** | ***Z*** | ***p*** | **CI low** | **CI high** | **Q** | **Q_p_** | **tau^2^** | **I^2^** | **H^2^** |
| --- | --- | --- | --- | --- | --- | --- | --- | --- | --- | --- | --- |
| Brooks et al. (2017) | 0.82 | 0.21 | 3.88 | 0.00 | 0.41 | 1.23 | 9.98 | 0.04 | 0.11 | 50.67 | 2.03 |
| Browning et al. (2020a) | 0.98 | 0.15 | 6.46 | 0.00 | 0.68 | 1.27 | 7.14 | 0.13 | 0.02 | 17.98 | 1.22 |
| Calogiuri et al. (2018) | 0.89 | 0.21 | 4.27 | 0.00 | 0.48 | 1.30 | 10.86 | 0.03 | 0.11 | 55.00 | 2.22 |
| Chirico and Gaggioli (2019) | 0.92 | 0.20 | 4.55 | 0.00 | 0.52 | 1.31 | 10.31 | 0.04 | 0.10 | 52.22 | 2.09 |
| Nukarinen et al. (2020) | 0.89 | 0.19 | 4.80 | 0.00 | 0.53 | 1.25 | 10.80 | 0.03 | 0.09 | 53.43 | 2.15 |
| Olafsdottir et al. (2018) | 0.75 | 0.13 | 5.77 | 0.00 | 0.49 | 1.00 | 3.85 | 0.43 | 0.00 | 1.98 | 1.02 |


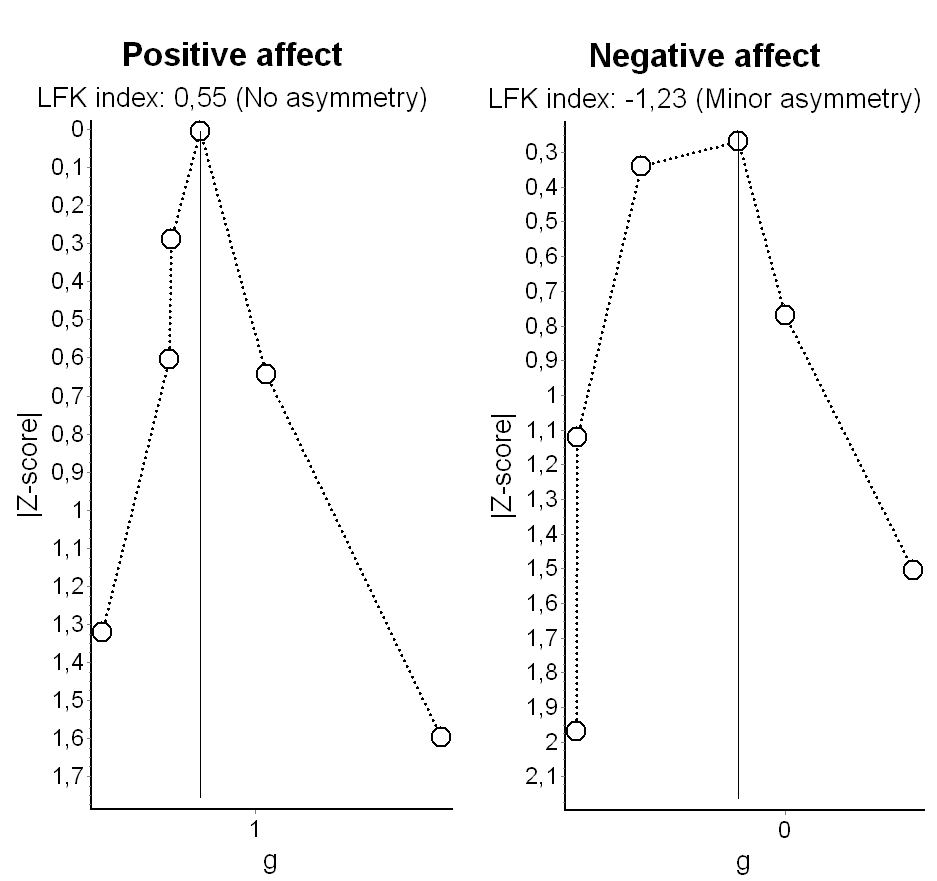


**Fig S1.** Doi plots for positive affect (left panel) and negative affect (right panel) change scores between exposures to actual versus simulated natural settings. For Positive affect, the distribution of the effect sizes (Hedge’s g) is symmetrical and the Luis Furuya-Kanamori (LFK) index is <|1|, which indicates no evidence of publication bias. For negative effect, there is only minor asymmetry and the LFK index is just above |1|, which indicates no evidence of concerning publication bias


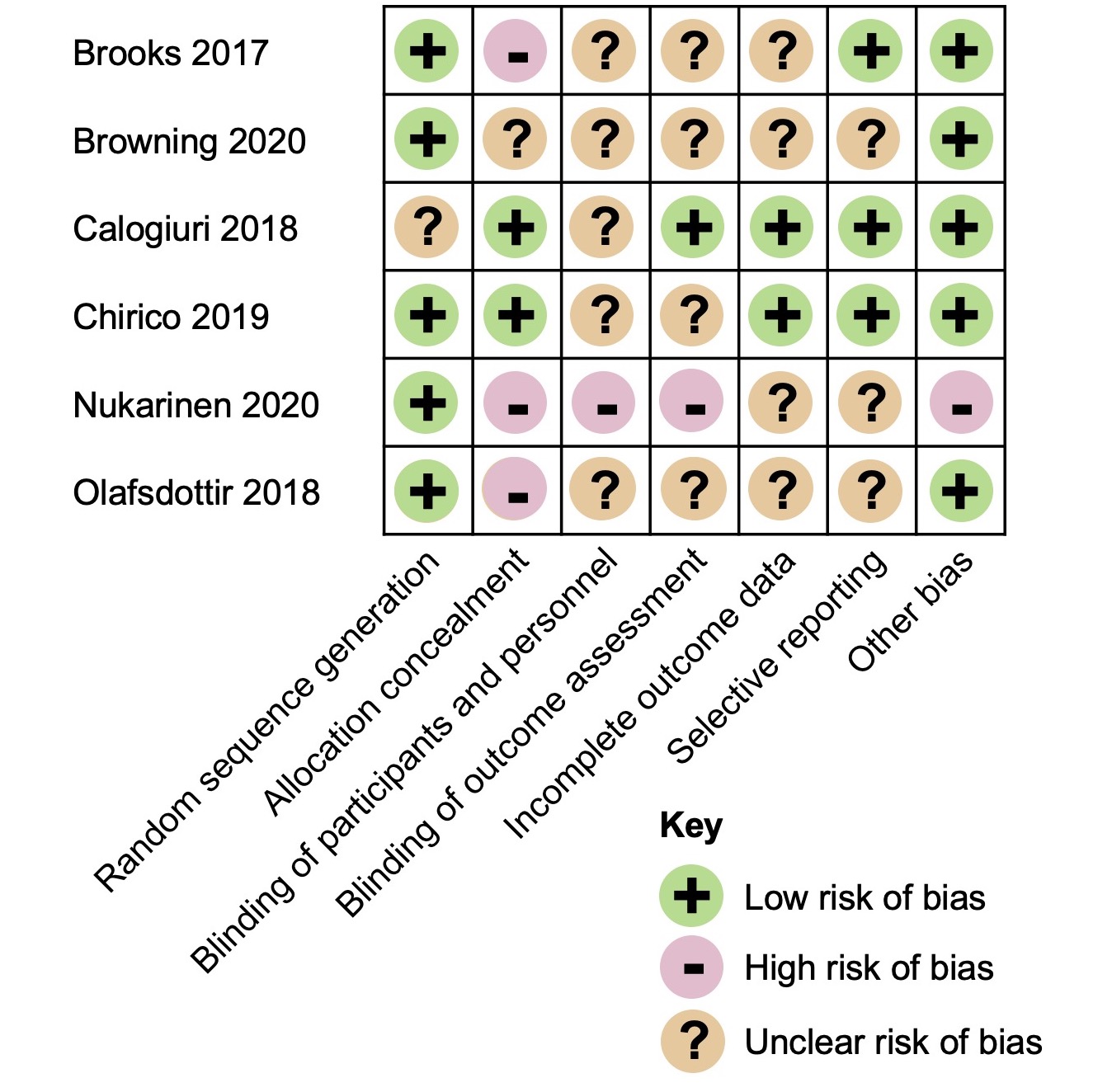


**Fig. S2.** Risk of bias for experimental studies that directly compared mood effects from exposure to simulated and actual natural settings.
